# Comparison of the Antibacterial Efficacy and Cytotoxicity of Herbal Nanoparticle-Incorporated Gels on Orthodontic Miniscrews: An In Vitro Study

**DOI:** 10.7759/cureus.98523

**Published:** 2025-12-05

**Authors:** Nivas Sundar, Raja A, Rehna Parvin N, Raja S, Srinidhi Ramasundaram

**Affiliations:** 1 Orthodontics and Dentofacial Orthopaedics, KSR Institute of Dental Science and Research, Namakkal, IND

**Keywords:** antibacterial efficacy, chlorhexidine, cinnamon nanoparticles, curcumin nanoparticles, cytotoxicity, herbal gels, orthodontic mini screws

## Abstract

Background

Orthodontic miniscrews are widely used as temporary anchorage miniscrews, but their prolonged stability can be compromised by microbial colonization and associated peri-implant inflammation. While chlorhexidine (CHX) is commonly used for its antimicrobial properties, concerns about its cytotoxicity have led to interest in alternative agents. Herbal extracts like curcumin and cinnamon possess better antimicrobial properties and biocompatibility, especially when formulated as nanoparticles with increased efficacy.

Aim

The aim of the study is to compare the antibacterial efficacy and cytotoxicity of herbal nanoparticle-incorporated gels on orthodontic miniscrews.

Materials and methods

In this study, gels containing curcumin and cinnamon nanoparticles were prepared, and 0.2% CHX gels were acquired. Antibacterial efficacy was assessed against *Streptococcus mutans* using the colony-forming unit (CFU) method. Cytotoxicity effects were assessed using the MTT (3-(4,5-dimethylthiazol-2-yl)-2,5-diphenyltetrazolium bromide) test. Orthodontic miniscrews were treated with each gel, and results were statistically analyzed using analysis of variance (ANOVA) and post hoc evaluation.

Result

Cinnamon nanoparticle-incorporated gels had a better antibacterial effect than curcumin nanoparticle-incorporated gel and CHX gel on orthodontic miniscrews. Cinnamon nanoparticle-incorporated gels and curcumin nanoparticle-incorporated gels exhibited the least cytotoxic effect than the CHX gel when applied on orthodontic miniscrews.

Conclusion

Cinnamon nanoparticle-incorporated gels coated on titanium miniscrews outperformed curcumin nanoparticle-incorporated gel and CHX gel in terms of both antibacterial efficacy and cytotoxicity.

## Introduction

Orthodontic miniscrews serve a vital role in orthodontic management by providing absolute or skeletal anchorage and simplifying biomechanics. The use of miniscrew implants as an adjunct for stability in the treatment of malocclusion has become widely accepted in recent years [1]. Kanomi in 1997 successfully used a mini-implant to intrude mandibular incisors [2].

In the initial days following miniscrew placement, patients often find it difficult to maintain appropriate oral hygiene due to pain, which encourages the growth of microorganisms. Microbial infections and inflammation of the adjacent hard and soft tissue lead to a failure rate of about 30% [3]. These failures are due to a bone defect around the neck of microimplants resulting from inflammation. Therefore, preventing inflammation around the screw implants is crucial for success [4]. Previous work has highlighted the role of microbial load in the stability and success of temporary anchorage devices (TADs), reporting that local inflammatory and microbiological factors negatively influence the outcome [5]. Systematic reviews have also shown that miniscrew performance influences patient-reported outcomes and overall treatment experience, further emphasizing the necessity for reliable antimicrobial strategies around TADs [6].

Biofilm formation has been prevented and managed with cetylpyridinium chloride, chlorhexidine (CHX), and fluoride mouthwashes. However, the majority of these substances are cytotoxic, can discolor teeth, and may even cause oral cancer, as is the case with ethanol, a common ingredient in mouthwashes [7].

CHX continues to be the benchmark antimicrobial material in dental healthcare practice and has been effectively used as a supportive measure in oral hygiene, for controlling plaque, reducing gingival inflammation, and minimizing bleeding [8]. It is formulated as gels and mouthwashes [9]. It is also used on a variety of medical instruments, including dental miniscrews, vascular access devices, and antibacterial dressings, and it outperforms povidone iodine in preventing surgical infections [10]. However, in vitro studies with CHX concentrations of 2% or more showed cytotoxic effects on the osteoblast, fibroblast, and myoblast. Reduced cell survival and the cessation of cell migration across all cell types were observed at concentrations as low as 0.002%. This indicates the significant cytotoxic potential of CHX at levels well below those used in clinical settings [11]. Other side effects of CHX-based products include taste loss, xerostomia, numbness, and allergic reactions [12].

This shows the need for an alternative with better antibacterial properties without any adverse effects. Herbal nanoparticles have been proven to exhibit better antibacterial properties. Cinnamon and curcumin might be considered a better alternative [13-15].

Based on the existing literature evidence, none of the studies have assessed the effectiveness of herbal nanoparticle-incorporated gels for reducing the biofilm formation on miniscrews and inflammation of the surrounding soft tissues. Therefore, the current study aimed to assess the antibacterial efficacy and cytotoxicity of cinnamon nanoparticle- and curcumin nanoparticle-incorporated gels with CHX gel.

The primary outcome of the present study is to evaluate the reduction in bacterial colony-forming units (CFU), while the secondary outcome aims to assess the percentage of cell viability using the 3-(4,5-dimethylthiazol-2-yl)-2,5-diphenyltetrazolium bromide (MTT) assay. The null hypothesis is that there is no significant difference in antibacterial efficacy and cytotoxicity between CHX gel, curcumin gel, and cinnamon gel on titanium (Ti) and stainless steel (SS) miniscrews.

## Materials and methods

The present study was conducted in the department of orthodontics and dentofacial orthopedics, KSR Institute of Dental Science and Research, Tiruchengode, and Saveetha Dental College and Hospital, Chennai. Institutional ethical clearance was obtained (378/KSRIDSR/IEC/2023).

Sample size determination

Sample size determination was performed with the G*Power software (version 3.1, Heinrich-Heine-University Düsseldorf, Düsseldorf, Germany), with 95% power and an alpha error of 5% (0.05) [5]. Normality of antibacterial efficacy (CFU) and cytotoxicity (% cell viability) data was assessed using the Shapiro-Wilk test. All groups were normally distributed (p > 0.05), allowing the use of parametric tests (one-way analysis of variance (ANOVA) with post hoc Scheffé’s test) for intergroup comparisons.

Orthodontic miniscrews

Twenty-one SS (SK Surgicals, Pune, India) and 21 Ti (SK Surgicals) miniscrews with dimensions of 1.3 × 6 mm were chosen for the study.

Cinnamon nanoparticles

A physical method known as ball milling for synthesizing nanoparticles using a top-down method was employed. Cinnamon bark powders were prepared using a mixture grinder. To obtain cinnamon nanoparticles, powdered samples were placed in a 50 mL SS jar with a 5 mm ball size. The weight ratio of balls to powder is 20:1, which was filled to about one-third of its volume capacity. During milling, the balls rotate horizontally at a constant speed of 500 rpm for about 10 hours, with the direction of the ball changing every 30 minutes. To avoid overheating, an air-cooling system was employed to maintain the equipment at a stable temperature of ~25°C, and the nanoparticles were prepared [16].

Curcumin nanoparticles

Curcumin nanoparticles were obtained from Sisco Research Laboratories Pvt. Ltd. (Mumbai, India). Nanoparticle-incorporated gels were formulated by incorporating these nanoparticles into the base polymers, as illustrated in Table 1.

**Table 1:** Formulation of curcumin and cinnamon nanoparticle-incorporated gels

SL. NO.	Ingredients	Role	Quantity
1	Triethanolamine	Gel base	2 g
2	Carboxymethylcellulose	Solidifying agent	3 g
3	Glycerin	Added preservative	1 mL

Weighed amounts of the ingredients were mixed together to obtain the base polymer. These measured quantities of cinnamon and curcumin were added to obtain nanoparticle-incorporated gels [17].

CHX gels

Commercially available CHX gels (Hexigel, ICPA Health Products Ltd, Mumbai, India) were utilized in the present study as a control.

Minimal inhibitory concentration

Minimal inhibitory concentration refers to the smallest concentration at which it is capable of inhibiting bacterial growth. Prior to gel formulation, the serial tube dilution technique was employed to obtain the minimal inhibitory concentration for both experimental nanoparticles. The minimal inhibitory concentrations for curcumin and cinnamon nanoparticles obtained in the current study were 128 µg/mL and 60-75 µg/mL, respectively.

*Streptococcus mutans* (ATCC 25175) was utilized in the current study for the evaluation of antibacterial efficacy. Their growth in the brain heart infusion broth medium was quantified. An overnight culture of bacteria was normalized to 1 × 108 to promote biofilm formation. The samples were then inoculated into 96-well bottom plates, and the bacteria were grown anaerobically at 37°C.

Coating of orthodontic miniscrews

All Ti and SS miniscrews were autoclaved and ultrasonically cleaned in distilled water for 10 minutes, followed by immersion in 70% ethanol for 15 minutes to remove surface contaminants. Specimens that were contaminated, damaged, or associated with contaminated culture medium were excluded from further processing. The screws were then air-dried under sterile conditions. Each miniscrew was coated using a standardized dip-coating technique. Screws assigned to the experimental groups were individually immersed in the respective nanoparticle-incorporated gels (cinnamon or curcumin) and rotated gently to ensure uniform distribution of the gel over the entire threaded surface. Excess gel was removed by allowing the samples to drain on sterile absorbent paper for one minute. The coated screws were then air-dried in a laminar airflow chamber for 30 minutes to stabilize the gel layer prior to antibacterial testing. Control group screws were coated similarly using commercial 0.2% CHX gel [5].

Assessment of antibacterial efficacy using CFU

**Figure 1:**
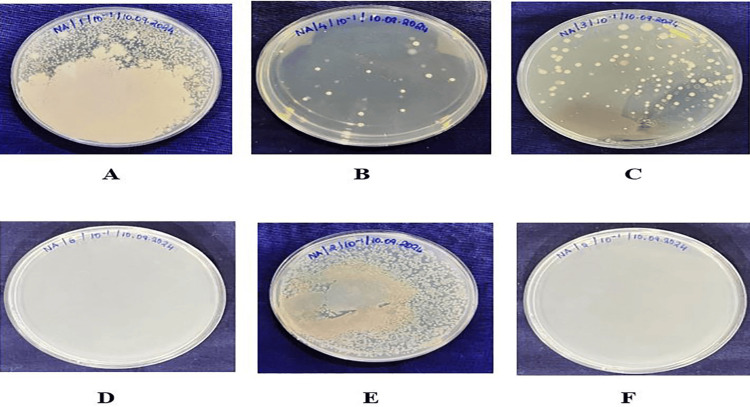
Assessment of antibacterial efficacy using colony-forming units (CFU) (A) CFU (Group 1: curcumin nanoparticle gel on stainless steel orthodontic miniscrews); (B) CFU (Group 2: curcumin nanoparticle gel on titanium orthodontic miniscrews); (C) CFU (Group 3: cinnamon nanoparticle gel on stainless steel orthodontic miniscrews); (D) CFU (Group 4: cinnamon nanoparticle gel on titanium orthodontic miniscrews); (E) CFU (Group 5: chlorhexidine gel on stainless steel orthodontic miniscrews); (F) CFU (Group 6: chlorhexidine gel on titanium orthodontic miniscrews)

Following bacterial inoculation, the coated specimens were immersed in culture well plates for 48 hours (Figure 1). After incubation, adherent *S. mutans* were detached into sterile saline, and a standard 10-fold serial dilution scheme (10⁻¹ to 10⁻³) was prepared by aseptically transferring 0.5 mL of the bacterial suspension into 4.5 mL of sterile saline, vortexing for 60 seconds, and repeating the same steps sequentially for each dilution tube. From the undiluted sample and each dilution, aliquots were plated for quantitative analysis. CFU/mL were counted using a digital colony counter, and colonies were identified based on macroscopic characteristics such as color and morphology, with Gram staining performed to confirm doubtful cases [18].

Cytotoxicity

To create a cell monolayer, 100 µL of human gingival fibroblast (HGF) cell suspension (4 × 104 cells/well) was pipetted into 96-well flat-bottom plates and maintained for 48 hours. After confirming monolayer growth under an inverted light microscope, the culture medium was removed. Twenty microliters of the respective gels and 100 µL of fresh culture medium were added to the respective wells.

Each eluate was incubated with 5% carbon dioxide (CO_2_) and 95% air at 37°C for 24 hours. After 24 hours, the toxic effect of gels was analyzed using the MTT assay [19].

Statistical analysis

Data were expressed as mean and standard deviation (SD). SPSS version 20.0 (IBM Corporation, Armonk, NY, US) was employed for statistical analysis. One-way ANOVA followed by post hoc Scheffé’s test was performed to assess statistical significance among the groups. A p-value of less than 0.05 was regarded as statistically significant at a 95% confidence interval.

## Results

Table 2 represents the mean antibacterial efficacy values between the experimental and control groups. Lower values of cell viability in the above table indicate more antibacterial efficacy calculated using one-way ANOVA. Among the six groups, Group 4 (cinnamon + Ti) was the most effective, and Group 1 (curcumin + SS) was the least effective. Between Ti and SS miniscrews, Ti exhibited better antibacterial activity irrespective of the incorporated gels.

**Table 2:** Mean antibacterial efficacy values between the experimental groups Group 1: curcumin nanoparticle gel on SS orthodontic miniscrews; Group 2: curcumin nanoparticle gel on Ti orthodontic miniscrews; Group 3: cinnamon nanoparticle gel on SS orthodontic miniscrews; Group 4: cinnamon nanoparticle gel on Ti orthodontic miniscrews; Group 5: CHX gel on SS orthodontic miniscrews; Group 6: CHX gel on Ti orthodontic miniscrews SS: stainless steel; Ti: titanium; CHX: chlorhexidine; SD: standard deviation

Groups	Antibacterial efficacy (mean ± SD)
Group 1 (curcumin + SS miniscrews)	241.86 ± 42.07
Group 2 (curcumin + Ti miniscrews)	72.85 ± 10.83
Group 3 (cinnamon + SS miniscrews)	130.86 ± 13.70
Group 4 (cinnamon + Ti miniscrews)	20.85 ± 5.33
Group 5 (CHX gel + SS miniscrews)	159.71 ± 8.65
Group 6 (CHX gel + Ti miniscrews)	77.14 ± 11.39

Table 3 represents the mean intergroup comparison values of antibacterial efficacy using post hoc Scheffe’s t-test expressed in mean and SD. There is no significant difference between Group 2 (curcumin + Ti) and Group 6 (CHX + Ti), and between Group 3 (cinnamon + SS) and Group 5 (CHX + SS). In contrast, discernible differences were noted comparing other groups regarding antibacterial efficacy (p < 0.05: statistically significant).

**Table 3:** Intergroup comparison of antibacterial efficacy between the experimental groups Group 1: curcumin nanoparticle gel on stainless steel orthodontic miniscrews; Group 2: curcumin nanoparticle gel on titanium orthodontic miniscrews; Group 3: cinnamon nanoparticle gel on stainless steel orthodontic miniscrews; Group 4: cinnamon nanoparticle gel on titanium orthodontic miniscrews; Group 5: chlorhexidine gel on stainless steel orthodontic miniscrews; Group 6: chlorhexidine gel on titanium orthodontic miniscrews *The corresponding value is statistically significant (p < 0.05) SD: standard deviation

Groups	Antibacterial efficacy (mean ± SD)	F-value	p-value
Group I with II	72.85 ± 10.83*	110.56	0.0001
Group I with III	130.86 ± 13.70*	113.59	0.0001
Group I with IV	20.85 ± 5.33*	109.28	0.0001
Group I with V	159.71 ± 8.65*	117.93	0.0001
Group I with VI	77.14 ± 11.39*	112.75	0.0001
Group II with III	130.86 ± 13.70*	109.56	0.0001
Group II with IV	20.85 ± 5.33*	97.76	0.002
Group II with V	159.71 ± 8.65*	125.76	0.0001
Group II with VI	77.14 ± 11.39	35.76	0.99
Group III with IV	20.85 ± 5.33*	117.35	0.0001
Group III with V	159.71 ± 8.6	29.57	0.210
Group III with VI	77.14 ± 11.39*	95.76	0.001
Group IV with V	159.71 ± 8.65*	116.79	0.0001
Group IV with VI	77.14 ± 11.39*	76.59	0.001
Group V with VI	77.14 ± 11.39*	126.59	0.0001

Table 4 represents the mean intergroup comparison values of cytotoxicity using one-way ANOVA followed by post hoc Scheffe’s t-test, expressed as mean ± SD. Significant differences between groups are indicated by p-values. Lower values indicate a more cytotoxic effect. More cytotoxic effects were seen with CHX, followed by the gel containing cinnamon and curcumin nanoparticles. No differences were noted in cytotoxicity between cinnamon and curcumin nanoparticle-incorporated gels. There was a marked difference in cytotoxicity between cinnamon and CHX gels and between CHX and curcumin gels.

**Table 4:** Mean values and post hoc values of cytotoxicity between the experimental groups p < 0.05: statistically significant difference compared to other groups SD: standard deviation; CHX: chlorhexidine

Groups	Percentage of cell viability (mean ± SD)	Intergroup comparison	F-value	p-value
Group 1 (curcumin)	95.24 ± 1.50	Group I with II	39.24	0.96
Group 2 (cinnamon)	94.65 ± 1.55	Group II with III	129.76	0.0001
Group 3 (CHX gel)	72.75 ± 6.39	Group III with I	116.34	0.0001

Table 5 represents the mean intergroup comparison values of antibacterial efficacy and cell viability using one-way ANOVA followed by post hoc Scheffe’s t-test, expressed as mean ± SD. Significant differences between groups are indicated by p-values.

**Table 5:** One-way ANOVA representing antibacterial efficacy and cell viability among the experimental and control groups ANOVA: analysis of variance

		Sum of squares	df	Mean square	F	Significance
Antibacterial efficacy	Between groups	212,703.357	5	42,540.671	110.561	0.000
	Within groups	13,851.714	36	384.770		
	Total	226,555.071	41			
Cell viability	Between groups	2,300.102	2	1,150.051	75.758	0.000
	Within groups	273.250	18	15.181		
	Total	2,573.352	20			

## Discussion

Miniscrew failure was attributed to a variety of factors. However, the presence of local factors with poor oral health maintenance was found to be the leading cause of miniscrew failure [3]. Incorporation of nanoparticles with antibacterial properties into orthodontic materials reduces plaque formation, thereby improving the retention and stability of mini-implants [5]. Various metal oxides, such as silver oxide, zinc oxide, Ti oxide, and copper oxide, were evaluated for antibacterial activity and cytotoxicity. However, some of these metal oxides were found to be cytotoxic [20,21]. Natural extracts demonstrated improved efficacy and reduced cytotoxicity profiles, suggesting viable substitutes for CHX in the management of oral and periodontal infections. Therefore, in the present study, we coated the surface of miniscrews with cinnamon and curcumin nanoparticle-incorporated gels and compared them with CHX gel to assess antibacterial efficacy and cytotoxicity.

The most common methods for incorporating nanoparticles onto Ti and SS surfaces were dip-coating, polydopamine-mediated coating, and hydrogel coating. The current study involves preparing hydrogels, coating them onto Ti and SS miniscrews, and assessing their antibacterial and cytotoxic properties. Hydrogels containing nanoparticles may provide an effective strategy for delivering antimicrobial agents in a long-term, controlled, and targeted manner. Several studies with long-term follow-up have found no significant difference in plaque and gingival indices between CHX gel and mouthwash. CHX mouthwash resulted in more tooth discoloration, and hence, CHX gel was utilized in the present study [22].

Different methods exist for evaluating antibacterial properties, each with its own merits and drawbacks. The CFU is the widely used gold standard method. The most significant advantage of CFU is the ability to determine bacterial counts at varying concentrations via serial dilutions. This method, however, counts only viable bacteria and eliminates dead bacteria and cellular debris [23]. The most commonly used method for evaluating cell viability is the MTT assay proposed by Mosmann et al. in 1983. It could be concluded that the activity of nicotinamide adenine dinucleotide phosphate (NADPH)-dependent cellular oxidoreductases is proportional to the count of viable cells [24].

From the results, we found that all three groups exhibited antibacterial properties, with better results shown by the herbal gel. Cinnamon nanoparticle-incorporated gels were found to have improved antibacterial properties, among all three groups. Cinnamon works through a variety of mechanisms. These include cell membrane damage, changes in lipid profile, inhibition of ATPases, membrane porins, motility, cell division, biofilm formation, and anti-quorum-sensing effects [25]. Current literature evidence reveals limited studies with cinnamon nanoparticles coated onto orthodontic miniscrews. Shafaee et al. [26] compared the cytotoxicity and antibacterial efficacy of cinnamon nanoparticles, copper oxide, and zinc oxide on glass ionomer cement as a luting agent for bands. They found that glass ionomers containing cinnamon nanoparticles outperformed other experimental groups in terms of antibacterial properties and toxicity. This is consistent with the results obtained from our study.

Curcumin nanoparticles were found to exhibit better antimicrobial properties compared to CHX gel. This was consistent with many studies. One such study by Le et al. concluded that the curcumin nanoemulsion-based gel had a softer texture and good antibacterial properties [27]. Ghavimi et al. also concluded in their study that the application of nanocurcumin gel within the implant fixture significantly reduced microbial counts in gingival crevicular fluid [28]. Alonso-Español et al. compared the antimicrobial activity of xanthohumol and curcumin on bacterial biofilms and confirmed their superior biofilm prevention properties [29]. The synergistic effect of curcumin-cinnamaldehyde hybrids (CCHs) over CHX was evaluated by Fiorillo et al. in terms of bacterial inhibition and compatibility. They inferred that CCHs outperformed CHX [22].

Between Ti and SS, nanoparticle-incorporated gels coated on Ti miniscrews showed better results than SS. A study done by Abdulhameed et al. comparing the surface roughness of different orthodontic archwires showed that Ti had better surface roughness than SS [30]. This might favor better retention of gels, thereby contributing to improved antimicrobial properties in the current study.

There is a significant difference between the experimental and control groups in terms of antibacterial efficacy and cytotoxicity; hence, the null hypothesis was rejected in the present study. Liu et al., in their study, confirmed that CHX was found to exhibit significant cytotoxicity even at a very low concentration when used for a longer period in clinical settings [11]. These results were in accordance with the current investigation, where CHX gel was found to have an increased cytotoxic effect over the tested herbal gels.

Within the limits of this in vitro study, it can be determined that cinnamon nanoparticle-incorporated gels have a good antibacterial effect and exhibit less cytotoxicity. We recommend that future studies should focus on the efficacy of herbal nanoparticle-incorporated gels against multi-species biofilms. In vivo investigations and long-term evaluation with multiple samples are necessary before translation to clinical practice.

Clinical implication

Cinnamon and curcumin nanoparticle gels may provide a safer, more biocompatible alternative to CHX for preventing microbial colonization on orthodontic miniscrews. Their use may reduce soft-tissue inflammation, improve miniscrew stability, and lower failure rates, especially in patients with poor oral hygiene.

## Conclusions

Cinnamon nanoparticle-incorporated gels had a better antibacterial effect than curcumin nanoparticle-incorporated gel and CHX gel on orthodontic miniscrews. There was a significant difference in the antibacterial efficacy among the three gels. Gels containing cinnamon nanoparticles exhibited superior antibacterial qualities when coated on Ti miniscrews compared to SS miniscrews. Cinnamon nanoparticle-incorporated gels and curcumin nanoparticle-incorporated gels exhibited the least cytotoxic effect than the CHX gel when applied on orthodontic miniscrews. There was a significant difference in the cytotoxicity between the herbal gels. Cinnamon nanoparticle-incorporated gels can be coated onto the miniscrews to inhibit bacterial growth. This will reduce the risk of miniscrews loosening due to inflammation and infection of the soft tissues.
